# Induction of Therapeutic Protection in an HPV16-Associated Mouse Tumor Model Through Targeting the Human Papillomavirus-16 E5 Protein to Dendritic Cells

**DOI:** 10.3389/fimmu.2021.593161

**Published:** 2021-02-25

**Authors:** Oscar Badillo-Godinez, Adolfo Pedroza-Saavedra, Veronica Valverde-Garduño, Victor Bermudez-Morales, Minerva Maldonado-Gama, Ricardo Leon-Letelier, Laura C. Bonifaz, Fernando Esquivel-Guadarrama, Lourdes Gutierrez-Xicotencatl

**Affiliations:** ^1^ Centro de Investigación Sobre Enfermedades Infecciosas, Instituto Nacional de Salud Pública, Cuernavaca, Mexico; ^2^ Hospital de Especialidades, Centro Médico Nacional Siglo XXI, Instituto Mexicano del Seguro Social, Mexico City, Mexico; ^3^ Laboratorio de Inmunología Viral, Facultad de Medicina, UAEM, Cuernavaca, Mexico

**Keywords:** HPV16-E5, anti-DEC-205, immunotherapy, dendritic cells, immune checkpoints, mouse tumor model

## Abstract

HPV E5 is an oncoprotein mainly expressed in premalignant lesions, which makes it an important target for a vaccine to prevent or cure cervical cancer (CC). In this study, we evaluated whether E5 targeted to DEC-205, present in dendritic cells (DCs), could induce a therapeutic protection against HPV16-induced tumor cells in a mouse model. The HPV-16 E5 (16E5) protein was cross-linked to a monoclonal antibody (mAb) specific to mouse DEC-205 (anti-DEC-205:16E5) or to an isotype control mAb (isotype:16E5). Rotavirus VP6 was cross-linked to the mouse anti-DEC-205 mAb (anti-DEC-205:VP6) as a non-specific antigen control. BALB/c mice were inoculated subcutaneously (s.c.) with the 16E5-expressing BMK-16/myc tumor cells, and 7 and 14 days later the mice were immunized s.c. with the conjugates, free 16E5 or PBS in the presence of adjuvant. Tumor growth was monitored to evaluate protection. A strong protective immune response against the tumor cells was induced when the mice were inoculated with the anti-DEC-205:16E5 conjugate, since 70% of the mice controlled the tumor growth and survived, whereas the remaining 30% developed tumors and died by day 72. In contrast, 100% of the mice in the control groups died by day 30. The anti-DEC-205:16E5 conjugate was found to induce 16E5-specific memory T cells, with a Th1/Th17 profile. Both CD4^+^ and CD8^+^ T cells contributed to the observed protection. Finally, treating mice that had developed tumors with an anti-PD-1 mAb, delayed the tumor growth for more than 20 days. These results show that targeting 16E5 to DEC-205, alone or combined with an immune checkpoint blockade, could be a promising protocol for the treatment of the early stages of HPV-associated cancer.

## Introduction

The human papillomavirus (HPV) is the most common agent of sexual transmission worldwide, affecting between 50 and 80% of the sexually active population. It is responsible for a third of virus-induced tumors and for 5% of all kinds of human cancers (1). The high-risk (HR)-HPVs are associated to CC, and the most frequently detected types are 16 and 18 (2).While the incidence of CC has declined due to prophylactic vaccination and effective screening programs, HPV-induced head and neck and anogenital tumors have increased (3–5).

The E5 oncoprotein from HPV16 (16E5) plays an important role both in the induction of cancer and in the metastasis process (6, 7). It is an 83-amino acid transmembrane protein, primarily localized in the intracellular membrane of the endoplasmic reticulum and the Golgi apparatus (8). The role of 16E5 has been studied mainly in the early stages of cervical carcinogenesis, since the expression of the E5 gene is apparently lost when the HPV DNA is integrated into the host cell genome (9, 10). However, a mixture of episomal and integrated stages of the HPV16 genomes has been observed in cervical lesions and cell lines from CC (11–15).

Due to the high prevalence of HPV infection worldwide, there is need for developing effective treatments against persistent infections and diseases associated with this virus. Although prophylactic vaccines have been effective in preventing HPV infection in healthy patients, their efficacy is reduced to 60% in individuals with previous infections or with HPV-associated lesions (16, 17). Unlike prophylactic vaccines that protect through the induction of neutralizing antibodies, therapeutic vaccines preferentially protect by stimulating a T-cell-mediated immune response that specifically eliminates the infected cells. Thus, E5 has been used as a therapeutic vaccine in mouse models. Liu and colleagues (18) showed that a strong protective immune response against 16E5-expressing tumor cells was induced in mice inoculated with a recombinant adenovirus codifying for E5. Similar results were obtained by Paolini et al. (19) using a DNA vaccine codifying for the 16E5 (19). In other studies, the inoculation of the E5 peptides 25–33 (20) or 29–37 (21) as epitopes for CD8^+^ T cells, in the presence of CpG ODN (agonist of TLR-9) as adjuvant, induced a strong response of IFN-γ-producing CD8^+^ T cells that protected against E5-expressing tumors.

The cellular immune response is tightly regulated, both by activation (co-stimulatory molecules) and by inhibitory signals (immune checkpoints). The checkpoints are responsible for maintaining tolerance by preventing autoimmunity. In this way, cancer cells escape from the T cell responses by expressing ligands to immune checkpoint molecules. Moreover, the tumor microenvironment can also induce these ligands in antigen-presenting cells (APC). PD-1 is the most studied immune checkpoint molecule present in T cells. The PD-1 protein is a negative regulator of T cell activity, and its interaction with the PD-L1 ligand, expressed on APC or cancer cells, induces a co-inhibitory signal that antagonizes TCR signaling; it also inhibits proliferation, cytokine production, and T cell survival (22, 23). Recently, mAb-based immunotherapy that blocks the PD-1/PD-L1 interaction has been used as a treatment to reactivate specific T cells against tumor antigens in cancer patients (24).

DCs are antigen presenting cells (APCs) that play a central role in the induction of T-cell responses. DCs express a large number of innate immune receptors associated to the cell membrane and to the cytosol, which allows them to efficiently sample the environment. When DCs encounter antigen in an inflammatory context, they carry out a maturation process that results in the over-regulation of co-stimulatory and MHC molecules and in the increase of antigen presentation in the context of MHC I or MHC II (25). In recent years, the study of the biology and function of DCs has identified different subpopulations of these cells in humans and mice (26). The DEC-205 endocytic receptor is a type C lectin receptor expressed in mouse and human DCs (27–30). This receptor is able to capture antigens, depositing them in rich MHC II late endosomes and favoring their cross-presentation in the context of MHC I, all of which results in the potent induction of CD4^+^ and CD8^+^ T cells (31, 32). Therefore, antigens have been targeted *in vivo* to the DEC-205 receptor by conjugation with a specific anti-DEC-205 mAb to stimulate antigen presentation by DCs. Moreover, potent protective responses against different infectious agents and cancer have been achieved when used together with a maturation stimulus (33–43). Thus, targeting tumor antigens to DCs through DEC-205 is a promising alternative for the treatment of malignant tumors.

The aim of this work was to evaluate whether targeting the 16E5 oncoprotein to DEC-205, present in DCs, could induce an effective protective immune response against a 16E5-expressing tumor cell line in a therapeutic model. We found that small amounts of 16E5, chemically conjugated to a rat anti-DEC-205 mAb and inoculated s.c. in mice with Poly I:C as adjuvant, induced a powerful specific protective response against the 16E5-expressing BMK-16/myc tumor cells. The procedure cured 70% of the experimental mice. This protection was found to be dependent on memory CD4^+^ and CD8^+^ T cells with a Th1/Th17 type phenotype. In addition, the administration of an anti-PD-1 mAb in mice with a retarded tumor growth (30%) caused an even greater delay of the process.

## Material and Methods

### Mice

Specific-pathogen-free, 6- to 8-week-old female BABL/c mice were provided by the animal house at the National Institute of Public Health (Cuernavaca, Morelos, Mexico). For experimental procedures, mice were housed in the same facility following the guidelines of the institutional Ethics Committee and the Mexican National Regulation on animal care and experimentation, under a standard light/dark cycle (12 h/12 h) and provided with food and water *ad libitum*.

### Cells

The BMK-16/myc cell line was generated by Crook and colleagues (44). Briefly, the baby BALB/c kidney cells (BMK), which are tumorigenic in syngeneic immunocompetent mice, were co-transfected to express the entire HPV16 genome and the murine c-*myc-1* gene (BMK-16/c-myc). MA-104 cells from Rhesus monkey kidney were purchased from ATCC (CRL-2378.1). Under *in vitro* conditions, the cells were grown in Dulbecco’s Modified Eagle’s Medium (DMEM) supplemented with 10% fetal bovine serum (FBS), 100 U/mL penicillin, 100 μg/mL streptomycin and 2 mM L-glutamine, and they were incubated at 37°C in humidified air containing 5% CO_2_. All cell culture reagents were from Invitrogen.

### Monoclonal Antibodies Production

The rat hybridomas producing the IgG2a mAb against mouse DEC-205 (NLDC-145) and the rat isotype control (IgG2a) (III-10) were donated by Dr. Ralph Steinman (Laboratory of Cellular Physiology and Immunology, The Rockefeller University, New York). The mouse hybridoma against Histidine tag (6His) (clone 2R-2A6) was generated at Dr. Gutierrez-Xicotencatl’s laboratory and characterized as IgG1 isotype (unpublished results).

For the production of the mAbs, the hybridomas were expanded in CD Hybridom serum-free medium supplemented with 0.2% FBS and 2 mM L-glutamine and purified as previously described (41). Briefly, the mAbs rich supernatants were precipitated with ammonium sulfate (50% w/v) for 1 h at room temperature, followed by centrifugation at 11,000 g for 15 min. The pellets containing the mAbs were re-suspended in one-tenth of the original volume with PBS/0.01% Tween-20 and dialyzed against PBS at 4°C for 16 h. Finally, the mAbs were purified by affinity chromatography with a sepharose-protein G column (Hiptrap, General Electric), according to the supplier’s protocol.

### Production and Purification of the HPV16 E5 Protein

The HPV16 *E5* gene was cloned and the protein was produced under *in vitro* conditions using the Rapid Translation System (RTS proteo Master, Roche) equipment, as previously described (45). Briefly, the 6His-tagged 16E5 recombinant protein (His-16E5) was produced in the RTS at 22°C for 16 h with continuous stirring, and it was purified by affinity chromatography on a Ni-NTA column (Qiagen), following the provider’s protocol. Fractions from the chromatography were analyzed by immune Western blot to verify the presence and identity of the His-16E5 protein. The anti-His mAb 2R-2A6 was used as first Ab, followed by a goat anti-mouse IgG polyclonal Ab conjugated to horseradish peroxidase (HRP). The antigen-Ab reaction was detected using the Western Lightning Chemiluminescence Reagent Plus (Perkin Elmer) and analyzed with the Odyssey Fc^®^ system. Positive fractions were mixed and protein concentration quantified by BCA kit (Pierce). The His-16E5 protein was aliquoted and stored at −80°C until use.

### Chemical Cross-Linking of mAbs With the Recombinant 16E5 Protein

The purified anti-DEC-205 and isotype control mAbs were conjugated with the purified recombinant 16E5 protein according to the previously described protocol (41). Briefly, 200 µg of mAb and 200 µg of 16E5 antigen were activated with succinimidyl-4- (N malemidomethyl) cyclohexane-1-carboxylate (SMCC) or with Traut’s reagent (2-iminothiolate or 2IT) (Pierce), respectively, following the manufacturer’s protocol. The reactions were placed separately in a 3-kDa molecular exclusion dialysis membrane and incubated for 2 h at 4°C against PBS. The activated mAb and the antigen were mixed and incubated for 16 h at 4°C, and the free antigen was removed by dialyzing with a 55-kDa molecular exclusion dialysis membrane against PBS. The conjugates were analyzed by SDS-PAGE and Western blot.

### Generation of Bone-Marrow DCs

DCs were derived *in vitro* from mouse bone-marrow as previously described (46). Bone-marrow cells were obtained from femurs and tibiae and cultivated in 2x10^6^ cells/100 mm^2^ bacteriological Petri dishes with RPMI medium supplemented with 10% FBS, 2 mM glutamine, 10 µg Gentamicin, 50 mM 2-mercaptoethanol (2-ME), and 250 U/ml of recombinant mouse GM-CSF (granulocyte-macrophage colony stimulating factor) (Invitrogen). Three days later, the cells were fed with the same RPMI supplemented GM-CSF medium. On days 6 and 9, half of the medium containing clusters of suspended cells was centrifuged at 600 g for 5 min and recovered cells were re-suspended in fresh RPMI supplemented GM-CSF medium and added back to the same culture. Finally, between days 10 and 12, the DCs-enriched suspension was collected.

### Cell Lysates

MA-104 cells and bone marrow-derived DCs were lysed with lysis buffer (10mM Hepes, 1.5 mM MgCl_2_, 150 mM NaCl, 2 mM EDTA, 2 mM EGTA, 0.5% Triton X-100, pH 7.2, and protease inhibitors from Roche) and incubated at 4°C for 30 min. Cell lysates were centrifuged at 13,000 g for 15 min at 4°C. The supernatants obtained were aliquoted and kept at −80°C until use.

### Western Blot for Detection of Anti-DEC-205 and 16E5 Protein

The mAb conjugates and cell lysates were separated on a 10% SDS-PAGE and transferred to a PROTEAN nitrocellulose membrane (Whatman International Ltd.), as previously described (47). The membrane was blocked with PBS-T buffer (0.05% Tween-20 in PBS) containing 10% w/v fat-free milk for 30 min at room temperature, with constant shaking. After blocking, the membrane was washed with PBS-T buffer and incubated with a rabbit anti-rat IgG polyclonal Ab (Sigma) in the presence of anti-DEC-205 or with the anti-His mouse mAb (2R-2A6) in the presence of 16E5 antigen diluted in PBS-T/5% fat-free milk for 16 h at 4°C, with constant shaking. Specific Ab binding was detected by using goat anti-rabbit IgG (for detection of anti-DEC-205) or goat anti-mouse IgG (for detection of 16E5 antigen) linked to HRP (DAKO), for 1 h at room temperature, and visualized by the Western Lightning Chemiluminescence Reagent Plus kit (Perkin Elmer). The images were captured using the Odyssey Fc^®^ system and analyzed with the Image Studio Lite 4.0.21 software.

### Immunizations and Tumor Generation

Groups of BALB/c mice were inoculated s.c. in the back with 5x10^5^ BMK-16/myc cells. Seven and fourteen days later the mice were immunized s.c. with anti-DEC-205:16E5, isotype:16E5 or anti-DEC-205:VP6 (as non-specific antigen control) with the equivalent of 5 µg of 16E5 or 1.5 µg of VP6, 5 µg of free 16E5 antigen, or PBS alone, all in the presence of 50 µg of Poly I:C (Amersham BioSciences) as adjuvant. Tumor growth was evaluated every 3 days by measuring the tumor volume with a caliper. All mice were sacrificed, and recorded as dead, when the tumor reached a volume between 500–700 mm^3^, unless otherwise stated. The efficacy of the different treatments was evaluated by survival curves.

### *In Vivo* Treatment With Specific mAbs

To determine the role of T cells in the anti-tumoral immune response after anti-DEC-205:16E5 therapeutic immunization, mice were inoculated intraperitoneally (i.p.) with 250 µg of the anti-CD8 mAb (TIB 105), 500 µg of the anti-CD4 mAb (GK1.5) or 500 µg of the isotype control mAb on days 17, 20, and 23 after the inoculation of tumor cells. Treatment of mice with these Abs rendered about 90% depletion of the specific spleen T cells, which was determined by flow cytometry (data not shown).

In the case of the anti-PD1 treatment, mice vaccinated with anti-DEC-205:16E5 in the therapeutic model were inoculated i.p. with 90 µg of anti-PD1 mAb (RMP1-14, Biolegend) or with the same amount of the isotype control on days 45, 48, 51, 54, and 57 after the inoculation of tumor cells. In all the treatments, tumor growth and mice survival were monitored as described above.

### Stimulation of Memory T Cells *In Vitro*

Mice were vaccinated with free E5, anti-DEC-205:16E5, or treated with PBS as control; 30 days after the inoculation of tumor cells, cells from the draining lymph nodes (DLNs) were obtained and stimulated *in vitro*. For this procedure, 2 x 10^5^ cells were cultured in 96-well plates (Invitrogen) with 200 µL of supplemented RPMI in the presence of anti-DEC-205:16E5 or anti-DEC-205:VP6, equivalent to 5 µg of antigen. As control, cells were cultured with medium alone. After 7 days of incubation at 37°C in a 5% CO_2_ atmosphere, cells were collected and analyzed by flow cytometry with a battery of mAbs specific for different memory T cell populations. For the expression of intracellular IFN-γ and IL-17, the cells were incubated with 1 µg/mL of Brefelding A (eBioscience) for 4 more hours, at the end of the 7-day incubation. Finally, the cells were collected and analyzed by flow cytometry.

### Isolation of Infiltrating Tumor Leukocytes

At the end of the tumor follow-up, the mice were sacrificed and the tumors dissected, cut in small pieces, placed in break-up medium [400U/mL collagen D (Roche) and 5 µg/mL DNAse I (Roche) in Hanks balanced solution (HBSS)] without calcium or magnesium, and incubated for 1 h at 37°C, mixing every 30 min to disaggregate the tumor and the tumor infiltrated cells. The enzyme activity was inhibited by adding stop medium (RPMI 10% FBS supplemented with 5 mM EDTA), and the disaggregated cells were clarified using a 70 µm filter (BD) and washed with RPMI 10% FBS by low centrifugation. The cell pellet was re-suspended in RPMI 10% FBS containing 20 µg/mL DNAse I (Roche) and incubated for 5 min on ice. Subsequently, cells were recovered by low centrifugation, re-suspended in Percoll (Sigma) gradient (40 to 90%), and centrifuged at 1,000 g for 30 min at room temperature. The leukocyte band was obtained from the gradient; the cells were washed once with PBS and finally re-suspended in RPMI 10% FBS medium. They were analyzed for viability using the trypan blue exclusion method in a Neubauer chamber. Afterwards, the cells were treated for flow cytometry analysis.

### Real-Time PCR

Total RNA was isolated from tumor tissue samples with Trizol reagent (Invitrogen) according to the manufacturer’s instructions. The Maxima First Strand Reverse Transcription kit (Thermo Scientific) was used for the synthesis of cDNA, following the provider’s instructions. Real-time PCR reactions were performed with the following primers: HPV16-E5; Forward 5’-AACATTACTGGCGTGCTT-3’, Reverse 5’-AGAGGCTGCTGTTATCCA-3’ (21); HPV16-E7; Forward 5’-ATTTGCAACCAGAGACAACTG-3’, Reverse 5’-CAATATTGTAATGGGCTCTGT-3’ (48); β-Actin; Forward 5’-GGCTGTATTCCCCTCCATCG-3’, Reverse 5-’CCAGTTGGTAACAATGCCATGT-3’ (49); c-Myc; Forward 5’-CGGACACACAACGTCTTGGAA-3’, Reverse 5’-AGGATGTAGGCGGTGGCTTTT-3’ (50); PD-L 1 Forward, 5′-GGTGCGGACTACAAGCGAAT-3′, Reverse, 5′-TTCATGCTCAGAAGTGGCTGG-3′; PD-1 Forward, 5′-AAATCGAGGAGAGCCCTGGA-3′, Reverse 5′-CATGCCTTGAAACCGGCCTT-3′ (51). Reactions were carried out under the following conditions: 94°C for 5 min followed by 40 cycles of denaturation at 94°C 30 s, alignment at 60°C for 30 s, and extension at 72°C for 30 s. The reactions were prepared in RNAse-free water with the SYBR Advantage qPCR Premix kit (Clontech) following the supplier’s specifications, and 0.4 µM of the specific pair of primers were used. The relative quantitation of each sample was calculated with the 2 [−ΔΔC (T)] formula. The levels of ß-actin mRNA were used to normalize the gene expression. All reactions were performed in duplicate.

### Flow Cytometry

For cell surface staining, the cells were collected, washed twice with Binding Buffer (BB; 0.01% Sodium azide, 2mM EDTA and 2% FBS in PBS) and incubated with BB plus 1% rabbit serum for 20 min at 4°C for Fc receptor blocking. Afterwards, the cells were re-suspended in BB with different mAbs at the appropriate concentration and incubated at 4°C for 20 min. The following mAbs from Biolegend were used: anti-CD11c-biotin (HL3), anti-DEC-205 (NLDC-145) anti-CD45-APC (30-F11), anti-CD4-APC-Cy7 (GK1.5), anti-CD8-FITC (53-6.7), anti-CD44-BV510 (IM7), anti-CD62L-PE-Cy7 (MEL-14), anti-CD45-PE-Cy7 (30-F11), anti-CD25-PerCP-Cy5.5 (3C7), anti-FoxP3-PE (MF-14), anti-PD1-APC (29F,1A12), anti-MHC-II-FITC (39-10-8), and anti-PD-L1-biotin (10F,9G2). The cells were stained with LIVE/DEAD Fixable Aqua (ThermoScientific) to analyze their viability. Finally, the cells were washed and fixed in 2% paraformaldehyde at room temperature for 20 min, and 10,000 cells per sample were acquired in a BD FACSCanto II flow cytometer (Beckton Dickinson) and analyzed with the FlowJo software (Tree Star, Inc.). When the first Ab was biotinylated, the cells were washed and incubated with STV- PE-Cy7/APC. For intracellular staining, the cells were permeabilized using the BD Biosciences’s Cytofix/Cytoperm kit (following the provider’s indications), incubated with the anti-IL-17-PE (TC11-18H10.1) and anti-IFN-γ-APC (XMG1.2) mAbs, and processed as indicated above. After the cells were labeled with the mAbs conjugated with 16E5, they were washed and incubated with the anti-His 2R-2A6 mAb followed by an anti-mouse IgG-Alexa 448 polyclonal Ab (poly 4073) (Biolegend). They were analyzed by flow cytometry as indicated above.

### Statistical Analysis

The Mann-Whitney U test was performed for the analysis of tumor growth to determine the statistical differences between two different treatments, as the Shapiro-Wilks test showed that the distribution of this data is non-parametric. A two-way ANOVA, followed by a Dunnett’s multiple comparisons test, was performed for the analysis of T-cell marker populations, and of cytokines vs. group treatments. In terms of the survival analysis, the Mantel-Cox log-rank test and the Kaplan Meier method were used to describe the efficacy of the treatments. *p* values < 0.05 were considered statistically significant. The statistical analysis was performed using the GraphPad Prism Software version 6.0 (San Diego, CA, USA) and STATA version 15.0.

## Results

### Production of Monoclonal Antibody Cross-Linked to 16E5 Oncoprotein

**Figure 1:**
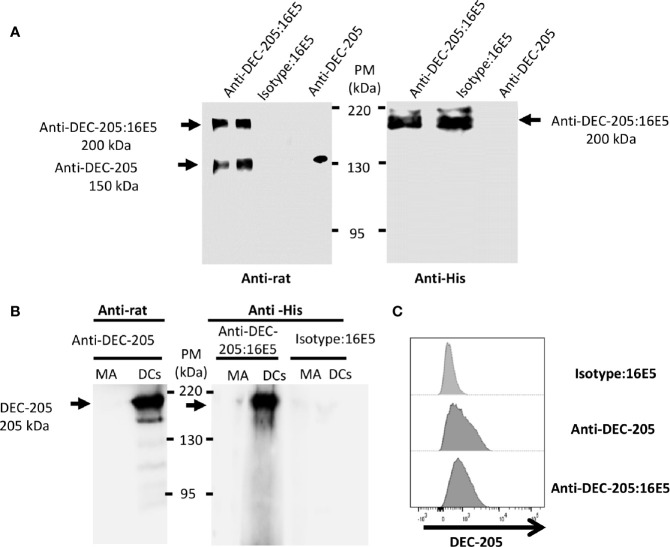
Analysis of the mAbs conjugated with antigen. The anti-DEC-205 mAbs and the isotype control were chemically cross-linked to 16E5 and characterized by Western blot and flow cytometry. **(A)** The conjugates were resolved in an 8% SDS-PAGE under non-reducing conditions and analyzed by Western blot. As control, non-conjugated anti-DEC-205 mAbs were used. The membrane was incubated with a rabbit anti-rat IgG polyclonal Ab, followed by a goat anti-rabbit IgG polyclonal Ab-HRP (*left panel*). To detect 16E5, a different membrane was incubated with an anti-His mAb, followed by a goat anti-mouse IgG polyclonal Ab-HRP (*right panel*). Signal was developed by chemiluminescence. **(B)** BM-derived DCs or MA-104 cells (MA) were lysed and 20 µg of total protein were developed in an 8% SDS-PAGE under non-reducing conditions and analyzed by Western blot. The membrane was incubated with the anti-DEC-205 mAb, followed by a rabbit anti-rat IgG polyclonal Ab-HRP (*left panel*). A different membrane was incubated with the anti-DEC-205:16E5 or isotype:16E5 conjugates, followed by the anti-His mAb and by a goat anti-mouse IgG polyclonal Ab-HRP (*right panel*). Signal was developed by chemiluminescence. **(C)** BM-derived DCs were stained with the anti-DEC-205 mAb, followed by a goat anti-rat IgG polyclonal Ab-FITC, or stained with the anti-DEC-205:16E5 or isotype:16E5 conjugates, followed by the anti-His mAb and a goat anti-mouse IgG polyclonal Ab-FITC. The cells were analyzed by flow cytometry.

The 16E5 protein was cross-linked with the anti-DEC-205 mAb or with the mAb III-10 as an isotype control for *in vivo* targeting to DCs, as indicated in *Material and Methods*. After the cross-linking process, the complexes were analyzed by Western blot using an anti-rat IgG Ab. The result showed that the cross-linking was not complete, since two bands were observed (**Figure 1A**, left panel): a 150-kDa one corresponding to free anti-DEC-205 mAb and a 200-kDa one corresponding to the complex consisting of anti-DEC-205 mAb and six molecules of the 16E5 oncoprotein (anti-DEC-205:16E5), calculated according to 16E5’s expected molecular weight (8.3 kDa). To corroborate that the 16E5 oncoprotein was present in the 200-kDa complex, a Western blot was carried out and developed with the anti-His mAb 2R-2A6 that recognizes the 6His tag present in the 16E5 oncoprotein (**Figure 1A**, right panel). The efficiency range of the conjugation was calculated to be around 70% in each preparation.

To determine if the specificity of the conjugated anti-DEC-205:16E5 was maintained, lysates of 12-day cultured DCs from mouse bone marrow (BM) were analyzed by Western blot using the anti-DEC-205 mAb alone (as positive control) followed by an anti-rat Ab, or the anti-DEC-205:16E5 or isotype:16E5 conjugates followed by the anti-His mAb and an anti-mouse IgG. Lysates of the simian epithelial cell line MA-104 were used as a negative control. With the anti-DEC-205 mAb alone and the anti-DEC-205:16E5 conjugate it was possible to recognize a 205-kDa band that corresponded to the expected molecular weight of the DEC-205 receptor in the DC lysates (**Figure 1B**, DCs left and right panels). No signal was detected with the isotype:16E5 conjugate (**Figure 1B**, right panel). To corroborate the specificity of the anti-DEC-205:16E5 conjugate, the mouse BM-derived DCs were stained with anti-CD11c and anti-DEC-205 mAbs followed by an anti-rat IgG Ab, or with the conjugates followed by an anti-His mAb and anti-mouse IgG; they were analyzed by flow cytometry. As expected, a positive signal was observed when the DCs were stained with the anti-DEC-205 mAb (**Figure 1C**), and a similar result was obtained when the anti-DEC-205:16E5 conjugate was used. Under the same conditions, the isotype:16E5 conjugate used as control did not display a positive signal. These results show that the anti-DEC-205 mAb conjugated to 16E5 maintained its specificity towards the DEC-205 receptor in DCs.

### Induction of Specific HPV-Tumor Protection by Targeting 16E5 Oncoprotein to DCs

**Figure 2:**
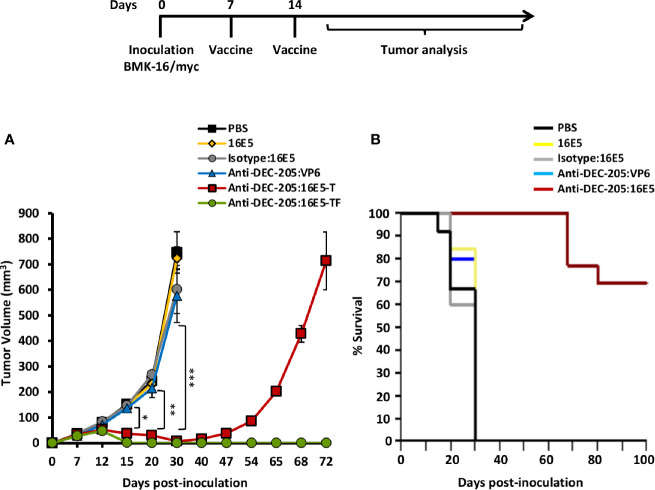
Targeting 16E5 to DCs induces tumor growth reduction and increases mice survival. BALB/c were inoculated s.c. with 5x10^5^ BMK-16/myc tumor cells and 7 and 14 days later immunized s.c. with anti-DEC-205:16E5, isotype:16E5, anti-DEC-205:VP6, free 16E5 or PBS in the presence of Poly I:C. Tumor growth **(A)** and mice survival **(B)** were followed up to 100 days. In the group immunized with DEC-205:16E5 two curves of tumor growth are shown, one corresponding to mice that developed tumors (T) (anti-DEC-205:16E5-T), and the other to mice that did not develop tumors (tumor-free; TF) (anti-DEC-205:16E5-TF). For the tumor growth graph data from one representative experiment (five mice per group) out of three is shown. For the survival curve data from three independent experiments (with a total of 12 mice per group) is shown. Vertical bars indicate standard deviation. Statistically significant differences are represented as *p* values (*< 0.05; **< 0.01; ***< 0.001).

To determine whether the anti-DEC-205:16E5 conjugate could induce a protective immune response against an HPV16-induced tumor, a therapeutic protocol was evaluated. For this, mice were first inoculated s.c. with the BMK-16/myc cells (that express E5, E6 and E7 from HPV16) for tumor induction. On days 7 and 14, different groups of mice were immunized s.c. with an amount of anti-DEC-205:16E5 conjugate equivalent to 5 μg of 16E5, in the presence of Poly I:C as adjuvant. The same conditions were used for the antigen control groups (isotype:16E5, free 16E5 or anti-DEC-205:VP6) and the PBS control. Tumor growth and mice survival were followed up for 72 and 100 days, respectively. The results showed that mice treated with isotype:16E5, free 16E5 or DEC-205:VP6 developed fast-growing tumors reaching a maximum tumor growth, which killed the mice by day 30, similarly to the control mice inoculated with PBS (**Figure 2A**). However, the mice treated with the anti-DEC-205:16E5 conjugate had an overall tumor reduction of 90% by day 30 compared to the control. After this period, a complete elimination of the tumor was achieved in 70% of the mice, which remained tumor-free for up to 100 days (**Figure 2B**), and 30% developed tumors and eventually died by day 72. The data show that targeting 16E5 to DCs in a therapeutic vaccination protocol induced a strong specific protection against an HPV16-induced tumor, compared to free 16E5 or 16E5 conjugated to an irrelevant mAb.

### Induction of Central Memory and Effector Memory-Specific T Cells Implicated in Protection by Targeting 16E5 to DCs

**Figure 3:**
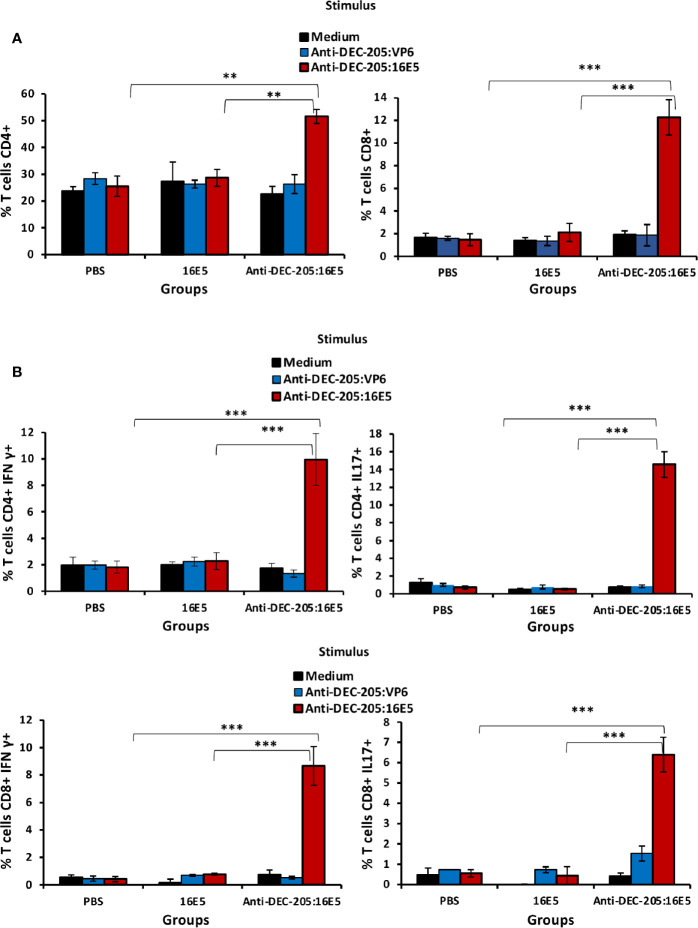
Targeting 16E5 to DCs induces antigen-specific T cells with a pro-inflammatory phenotype in DLNs. Mice were immunized with anti-DEC-205:16E5, free 16E5 or PBS in the presence of Poly I:C, using the therapeutic model described above. After 30 days of tumor cell inoculation, cells from the DLNs were stimulated *in vitro* with anti-DEC-205:16E5, anti-DEC-205:VP6 (as irrelevant antigen) or medium alone. After 7 days of stimulation, cells were collected, stained with a battery of mAbs specific for different T cell markers and cytokines coupled with fluorochromes, and analyzed by flow cytometry. **(A)** Percentage of CD4^+^ (*left panel*) or CD8^+^ (*right panel*) T cells. **(B)** Percentage of CD4^+^ IFN-γ^+^ and CD8^+^ IFN-γ^+^ T cells (*left panels*) and percentage of CD4^+^ IL-17^+^ and CD8^+^ IL-17^+^ T cells (*right panels*). Data from one representative experiment (3 mice per group) out of two is shown. Vertical bars indicate standard deviation. Statistically significant differences are represented as p values (**< 0.01; ***< 0.001).

**Figure 4:**
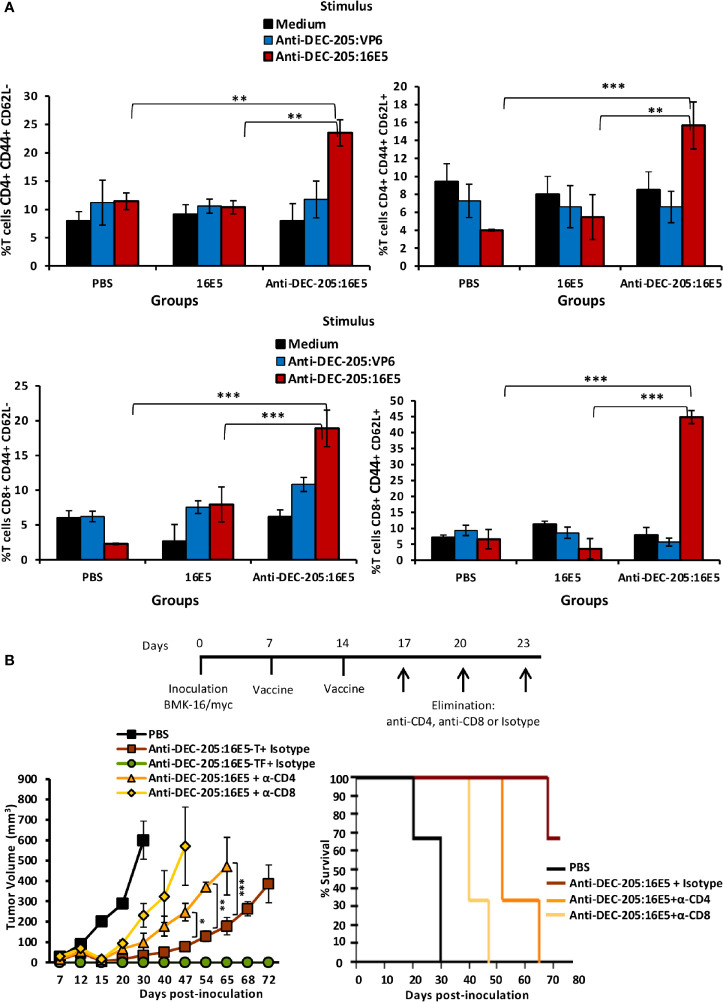
16E5-specific memory T cells play an important role in protecting against tumor cells. **(A)** Mice immunization was performed as indicated in **Figure 2** with anti-DEC-205:16E5, anti-DEC-205:VP6 or PBS. *In vitro* antigen stimulation of cells from DLNs and T cell staining was performed as indicated in **Figure 3**. The *left panels* show the percentage of CD4^+^ CD62L^-^ and CD8^+^ CD62L^-^ T cells (effector memory) and *right panels* show the percentage of CD4^+^ CD62L^+^ and CD8^+^ CD62L^+^ T cells (central memory). Data from one representative experiment (3 mice per group) out of three is shown. **(B)** Mice were inoculated with 5x10^5^ BMK-16/myc tumor cells and immunized with anti-DEC-205:16E5 or PBS in the presence of Poly I:C, as indicated in **Figure 2**. On the indicated days, groups of mice immunized with anti-DEC-205:16E5 were inoculated i.p. with anti-CD4-, anti-CD8 mAbs, or mAb III-10 as isotype control. Tumor growth (*left panel*) and mice survival (*right panel*) were followed up for 72 days. In the tumor growth panel, the mice treated with anti-DEC-205:16E5 and the mAb isotype control were divided in mice that developed tumors (anti-DEC-205:16E5-T) and mice that remained tumor-free (anti-DEC-205:16E5-TF). Data from one representative experiment (four mice per group) out of two is shown. Vertical bars indicate standard deviation. Statistically significant differences are represented as *p* values (*< 0.05; **< 0.01; ***< 0.001).

It is known that both CD4^+^ and CD8^+^ T cells may play an important role in protecting against malignant tumors. To evaluate the T cell response induced by anti-DEC-205:16E5 vaccination, the mice were inoculated with BMK-16/myc tumor cells and treated with the anti-DEC-205:16E5 conjugate, free 16E5 or PBS plus adjuvant, using the protocol previously described. At day 30, cells from DLNs were isolated and stimulated for 7 days under *in vitro* culture conditions with anti-DEC-205:16E5, anti-DEC-205:VP6 (VP6 as non-specific antigen), or RPMI medium alone as control. The memory T cell response against 16E5 was analyzed by flow cytometry using fluorescent mAbs. It is important to mention that even though at day 30 the tumors of mice immunized with the anti-DEC-205:16E5 conjugate were very small (˜ 20 mm^3^) (**Figure 2A**), they were obtained and analyzed. The results showed that only the group of mice immunized with the anti-DEC-205:16E5 conjugate were able to generate a memory CD4^+^ (2-fold increment) and CD8^+^ (6-fold increment) T cell response against 16E5 in DLNs, compared to the control groups (**Figure 3A** and **Supplementary Figure 1A**). When the intracellular pro-inflammatory cytokines IFN-γ and IL-17 were analyzed, memory CD4^+^ and CD8^+^ T cells stimulated with anti-DEC-205:16E5 were found to synthesize large amounts (4.5 – 14-fold increment) of those cytokines in comparison to the control groups (**Figure 3B** and **Supplementary Figure 1B**). This response involved at least two types of memory CD4^+^ and CD8^+^ T cells: effector memory T cells (CD44^+^ CD62L^-^) (**Figure 4A**, left panels, and **Supplementary Figure 1C**) and central memory T cells (CD44^+^ CD62L^+^) (**Figure 4A**, right panels, and **Supplementary Figure 1C**). These results showed that vaccination with the anti-DEC-205:16E5 conjugate generated both central and effector memory CD4^+^ and CD8^+^ T cells with a combined Th1 and Th17-type phenotype.

To determine whether the protection induced by the anti-DEC-205:16E5 conjugate was dependent on memory CD4^+^ and/or CD8^+^ T cells, the mice were inoculated with BMK-16/myc cells and treated with the anti-DEC-205:16E5 conjugate or PBS plus adjuvant using the therapeutic protocol described above. The mice inoculated with the anti-DEC-205:16E5 conjugate were divided into three groups and inoculated i.p. on days 17, 20 and 23 with an anti-CD4, an anti-CD8 mAb, or a control isotype, in order to deplete the CD4^+^ or the CD8^+^ T cell populations. As expected, the mice inoculated with PBS only reached the maximum tumor growth by day 30 (**Figure 4B**, left panel) with a mortality rate of 100%, whereas the mice vaccinated with the anti-DEC-205:16E5 conjugate and treated with the isotype control mAb showed a delayed tumor growth up to day 72 and a mortality rate of 30%. In contrast, CD4^+^ and CD8^+^-depleted mice showed an accelerated tumor growth, reaching a maximum growth by days 65 and 47, respectively, and a mortality rate of 100% in both treatments (**Figure 4B**, right panel). This result showed that the protection induced by the anti-DEC-205:16E5 conjugate was dependent on both memory CD4^+^ and CD8^+^ T cells; however, the CD8^+^ T-cell population appeared to be the most important one in the process of eliminating tumor cells.

### Regulation of the Anti-Tumor Immune Response

**Figure 5:**
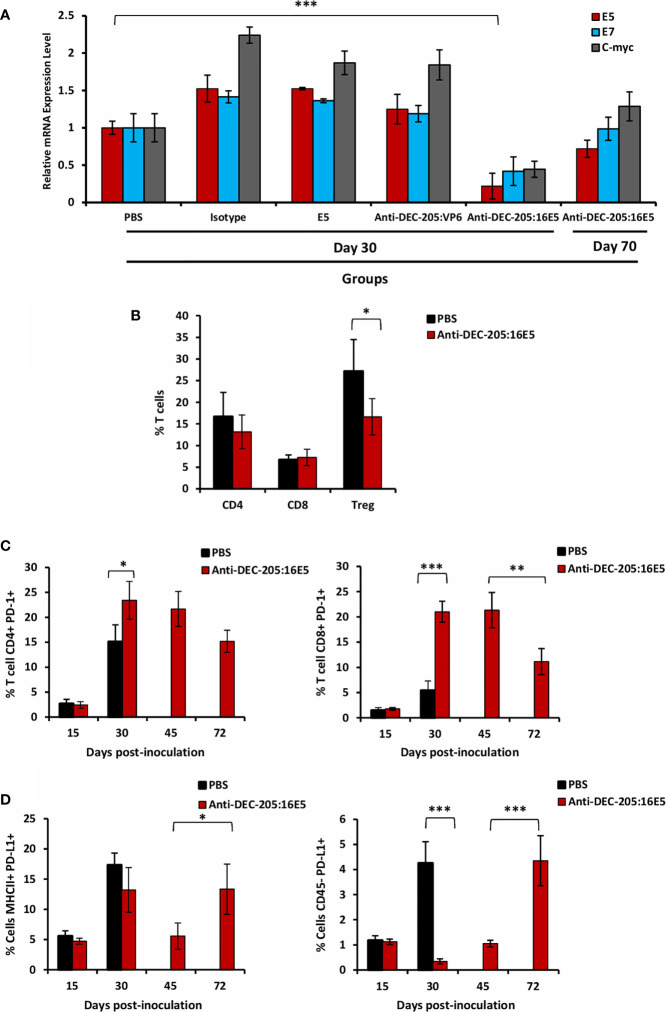
Effect of targeting 16E5 to DCs in the anti-tumor immune response. **(A)** Mice were inoculated with 5x10^5^ BMK-16/myc tumor cells and immunized s.c. with anti-DEC-205:16E5, isotype:16E5, anti-DEC-205:VP6, free 16E5 or PBS in the presence of Poly I:C, following the therapeutic model. Thirty and 70 days after tumor cell inoculation, the tumor was obtained and total RNA isolated. The expression levels of *E5, E7* and *c-myc* were analyzed by qRT-PCR and normalized with the expression levels of *β-actin*. A representative experiment out of two is shown. **(B)** Mice were inoculated as in A with anti-DEC-205:16E5 or PBS. Thirty days after tumor cell inoculation, TILs from tumors were obtained and stained with different mAbs coupled to fluorochromes and analyzed by flow cytometry. The percentages of CD4^+^ and CD8^+^ T cells as well as of CD4^+^ CD25^+^ Foxp3^+^ (Treg) are shown. **(C)** Mice were inoculated as in **(B)** Fifteen, 30, 45 and 72 days after tumor cell inoculation, TILs were obtained and stained with different mAbs coupled to fluorochromes and analyzed by flow cytometry. The percentages of CD4^+^ PD-1^+^ (*left panel*) and of CD8^+^ PD-1^+^ (*right panel*) T cells are shown. **(D)** Mice were inoculated as in **(C)** Fifteen, 30, 45, and 72 days after tumor cell inoculation, the cells from the disaggregated tumors were stained with different mAbs coupled to fluorochromes and analyzed by flow cytometry. The percentages of MHC II^+^ PD-L1^+^ (*left panel*) and of CD45^-^ PD-L1^+^ (*right panel*) cells are shown. Data from one representative experiment (three mice per group) out of two is shown for **(B–D)** Vertical bars indicate standard deviation. Statistically significant differences are represented as *p* values (*< 0.05; **< 0.01; ***< 0.001).

As stated before, 30% of the mice immunized with the anti-DEC-205:16E5 conjugate developed delayed tumor growth, which indicates that the tumor cells somehow escaped the 16E5-specific effector memory T cells. One of the strategies of the tumor cells to escape the immune response is the down-regulation of the tumor-associated antigens. Since in our model the target antigen is the 16E5 protein, at day 30 we analyzed by qPCR the level of its transcripts in tumor cells from the different groups. In the case of mice vaccinated with the anti-DEC-205:16E5 conjugate, tumor samples were obtained from the surviving mice at days 30 and 70 post-tumor inoculation. Tumor cells from mice treated with isotype:16E5, free 16E5, and anti-DEC-205:VP6 showed a 30 to 50% increase in 16E5 transcript levels compared to tumor cells from mice inoculated with PBS (**Figure 5A**). However, the 16E5 transcript levels from tumor cells from mice treated with anti-DEC-205:16E5 at day 30 showed a 70% decrease compared with tumor cells from mice treated with PBS, but surprisingly, by day 70, the 16E5 transcript levels of the mice that developed tumors increased to levels close to those found in PBS-treated mice (**Figure 5A**). When the E7 (under the control of the same promoter as E5) and the *c-myc* (regulated by a different promoter) transcript levels (44) were evaluated, similar results were obtained in all of the groups (**Figure 5A**). These results suggest that, in the absence of a memory T-cell response, the tumor cells maintain high levels of 16E5, the target tumor antigen. However, under T cell pressure, the escaped tumor cells drastically reduce the expression of the antigen to minimum levels, albeit enough to maintain the tumorigenic potential. This selective pressure down-regulates genes, such as E7, and others under a different promoter, such as *c-myc*.

Another possible strategy used by tumor cells to escape the immune response is regulating the effector functions of the tumor infiltrating leukocytes (TILs). In order to determine whether this was the case in our model, TILs were isolated from 30-day tumors of PBS or anti-DEC-205:16E5 conjugate-treated mice and analyzed by flow cytometry using a panel of T cell markers. No differences were found in the percentages of TIL CD4^+^ and CD8^+^ T cells between PBS and anti-DEC-205:16E5-treated mice (**Figure 5B**, and **Supplementary Figure 2A**). However, when the regulatory T cells (Treg, Foxp3^+^) were analyzed, they had decreased approximately 10% in the anti-DEC-205:16E5-treated mice compared to the control PBS-treated mice (**Figure 5B**, and **Supplementary Figure 2A**).

On the other hand, the expression of receptor PD-1 and its ligand PD-L1 have been related to the regulation of the T cells effector functions intratumorally. Thus, we first evaluated the expression levels of PD-1 on the surface of TIL T cells in tumors from PBS-treated mice, obtained at days 15 and 30, and from anti-DEC-205:16E5 conjugate-treated mice, obtained at days 15, 30, 45 and 72 (the last two from mice that developed tumors). The results showed that at day 15 there was a very low percentage of PD-1-expressing CD4^+^ and CD8^+^ T cells in both treatments (**Figure 5C** and **Supplementary Figure 2B**). However, by day 30 there was a dramatic increase of the percentage of CD4^+^ PD-1^+^ T cells in both treatments, although their presence in anti-DEC-205:16E5-treated mice was 7% higher than in PBS-treated mice, which was statistically significant (**Figure 5C**, left panel). In contrast, the CD8^+^ PD-1^+^ T cell percentage increased 15% by day 30 in the anti-DEC-205:16E5-treated mice compared with the PBS-treated mice (**Figure 5C**, right panel). Also, in the latter group, both the CD4^+^ and the CD8^+^ PD-1^+^ T cells remained at similar levels by day 45, but were reduced between 30% and 50% by day 72, respectively (**Figure 5C**). When the expression of PD-L1 was evaluated in intratumoral antigen-presenting cells (MHC-II^+^), the level of PD-L1^+^ cells was found to be around 5% on day 15, and it had increased somewhere between 12% and18% by day 30 in both treatments. Furthermore, the cells from the anti-DEC-205:16E5-treated mice reduced the expression of PD-L1 to basal levels by day 45, and it returned to similar levels as day 30 by day 72 (**Figure 5D**, left graph and **Supplementary Figures 3A, B**). It has been reported that tumor cells can also express PD-L1; in fact, the BMK-16/myc cells used here expressed detectable levels of PD-L1 in culture (data not shown). When the expression of PD-L1 was analyzed in tumor cells (CD45^-^ cells) isolated from digested tumor, the level of PD-L1 was found to be around 1% on day 15 in both treatments, and in the case of the PBS-treated mice, the level increased up to 5% by day 30. On the other hand, the anti-DEC-205:16E5-treated mice showed only background levels of PD-L1 on days 30 and 45, but a sharp increase to 4% was observed by day 72 (**Figure 5D**, right panel and **Supplementary Figures 3A, C**). Altogether, these results suggest that the tumor induced a regulatory environment as well as a non-functional state of T cells to evade immune attack. In the case of the anti-DEC-205:16E5 conjugate treatment, a down-regulation of the associated tumor antigen (16E5) during the early stages might have also contributed to the evasion of the T cell response.

### Increase in the Survival of Mice Vaccinated With the Anti-DEC-205:16E5 Conjugate by Blockage of the PD-1 Receptor

**Figure 6:**
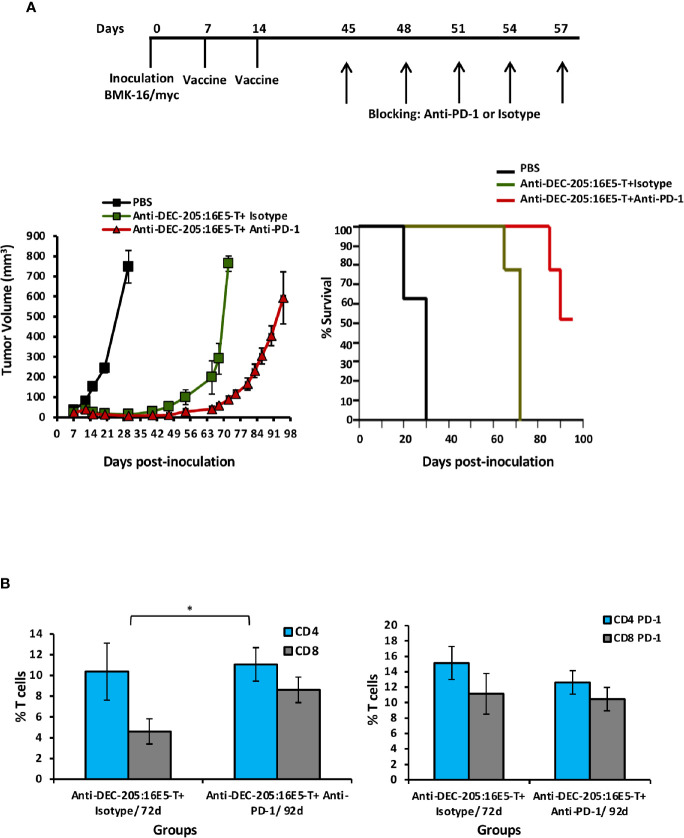
Targeting 16E5 to DCs together with anti-PD-1 immunotherapy improves the immune response against the tumor. **(A)** Mice immunization was performed as indicated in **Figure 2** with anti-DEC-205:16E5 or PBS, using the therapeutic model. Mice that developed tumors (30%) after vaccination with the anti-DEC-205:16E5 conjugate (anti-DEC-205:16E5T), were inoculated i.p. with the anti-PD-1 mAb or the mAb III-10 as isotype control on the indicated days. Tumor growth (*left panel*) and mice survival (*right panel*) were followed for up to 100 days. Data from one experiment with five mice per group is shown. **(B)** Seventy-two and 92 days after tumor cell inoculation, TILs were obtained and stained with different mAbs coupled to fluorochromes and analyzed by flow cytometry. The percentages of CD4^+^ and CD8^+^ (*left panel*) and of CD4^+^ PD-1^+^ and CD8^+^ PD-1^+^ (*right panel*) T cells are shown. Data from one experiment with three mice per group is shown. Vertical bars indicate standard deviation. Statistically significant differences are represented as *p* values (*< 0.05).

Considering that an important percentage of both CD4^+^ and CD8^+^ tumor-infiltrating T cells expressed PD-1 in the anti-DEC-205:16E5-treated mice, we tested whether an anti-PD-1 blocking mAb could increase mice survival. Thus, mice that developed tumors (30%) after vaccination with the anti-DEC-205:16E5 conjugate, according to the therapeutic protocol, were inoculated i.p. with the anti-PD-1 mAb rpm-1-14 or with the isotype control III-10 on days 45, 48, 51, 54 and 57. As control of tumor growth, non-vaccinated mice were inoculated with tumor cells. As expected, mice vaccinated with the anti-DEC-205:16E5 conjugate and treated with the mAb isotype control died by day 72. However, the treatment with the anti-PD-1 blocking mAb increased mice survival by 23 days (mice were sacrificed on day 95) (**Figure 6A**). The mAb isotype control-treated group displayed a 2:1 proportion of intratumoral CD4^+^: CD8^+^ T cells by day 72. However, on day 92, the anti-PD-1 mAb-treated mice showed a balanced proportion of these cells (**Figure 6B**, left graph), with similar levels of CD4^+^ T cells but higher levels of CD8^+^ T cells compared to the isotype-treated group. On the other hand, the proportion of CD4^+^ PD-1^+^ and CD8^+^ PD-1^+^ T cells was similar in both groups (**Figure 6B**, right panel). This data show that blocking the interaction of intratumoral PD-1^+^ T cells and PD-L1^+^ APC resulted in increased mice survival, most likely due to the rescue of effector T cells.

## Discussion

Even though there are two anti-HPV prophylactic vaccines in the market, this infection still causes around 5% of all cancers in the human population. E5 is an oncoprotein that is mainly expressed in the early stages of an HPV infection, so it can be a suitable target for preventing or eliminating HPV-induced tumor cells. Previous reports have shown that E5 and E5-derived synthetic peptides can induce protection against E5-expressing tumors (18–21). In this study, we intended to determine whether E5 targeted to DEC-205 present in DCs could induce protection against E5-expressing tumor cells in a BALB/c mouse model, in a therapeutic model. A protective immune response against the HPV16 E5-expressing BMK-16/myc tumor cells was found to be induced when mice were inoculated with the anti-DEC-205:16E5 conjugate (equivalent to 5 µg of E5). Seventy percent of the mice controlled the growth of the tumor and survived, and the remaining 30% developed tumors and died by day 72 post-tumor cells challenge. In contrast, 100% of the mice in the control groups died by day 30. The protection generated by the vaccine was T-cell dependent, and the treatment with an anti-PD-1 mAb in mice that developed tumors delayed tumor growth for more than 20 days.

We determined that the protection induced against the 16E5-expressing tumor cells was antigen-specific and dependent on the targeting of this protein to DEC-205, because mice immunized with the other treatments (isotype:16E5 conjugate, anti-DEC-205:VP6 conjugate, or 5 µg of free 16E5) did not show any protection, compared to the control group inoculated with adjuvant only. This is supported by the fact that the cross-linking process did not alter the recognition of DEC-205. The anti-DEC-205:16E5 conjugate was able to recognize the DEC-205 molecule present in DCs from the bone marrow (shown by immunofluorescence and Western blot), which validates the results obtained in the immunization protocols. Similar results have been previously reported using other viral antigens (39, 41, 52).

T cells, especially the CD8 subtype as direct effectors, play an important role in eliminating transformed tumor cells. However, CD4^+^ T cells are also important, since they induce efficient CD8 T cell responses (53) and, in some instances, act as direct effector cells (54). In fact, we found, E5-specific memory CD4^+^ and CD8^+^ T cells with a Th1/Th17 type phenotype in DLN from mice immunized with the anti-DEC-205:16E5 conjugate. The importance of these cells in the protection observed in mice immunized with the anti-DEC-205:16E5 conjugate was confirmed when they were depleted *in vivo* with specific mAbs, causing the tumors grew faster and leading to the death of 100% of the mice, compared to a mortality rate of 30% in the non-depleted control group. However, the role of CD8^+^ T cells appears to be more determinant in terms of protection, since the CD8^+^ T cell-depleted mice died 18 days earlier than the CD4^+^ T cell-depleted mice. As indicated above, the protective role of the CD4^+^ T cells in our model could consist in their ability to induce an effective activation of CD8^+^ T cells and/or in their activity as direct effectors, since they were found infiltrating the tumor. Further studies are necessary to elucidate their function in this model.

DCs play a fundamental role in inducing T cell responses, so targeting antigens to the DEC-205 present mainly in these cells has been a very successful strategy to potentiate antigen-specific T cell activity in cancer and infectious models (41, 43, 55). Our results regarding the 16E5-expressing tumor cells corroborate those findings. Although we did not determine the mechanism by which the protective 16E5-specific memory T cells were induced, previous studies have provided some evidence. Bonifaz and colleagues (2004) showed that when OT-I and OT-II mice were immunized s.c. with anti-DEC-205-Ovoalbumin (OVA), a potent anti-OVA T-cell response took place in DLNs and other distant LNs (31). When anti-DEC-205:fluoroscein was inoculated, the predominantly labeled cells were CD8^+^ DCs (cDC-1). These results suggest that the Ab-antigen conjugates are distributed locally and systemically in different LNs where CD8^+^ DC-expressing DEC-205 captures and internalizes them. Afterwards, the DCs present the antigenic peptides to the CD4^+^ T cells through the exogenous pathway and to the CD8^+^ T cells by cross-presentation. More recent studies have confirmed that cDC-1 play an important role in tumor immunity (56). Therefore, it is very likely that in our model, the CD8^+^ DEC-205 DCs present in secondary lymphoid organs and targeted with the anti-DEC-205:16E5 conjugate were responsible for inducing the specific memory CD8^+^ and CD4^+^ T cells, which, in the presence of the Poly I:C as adjuvant, they are driven to the Th1/Th17 phenotype. Moreover, Poly I:C has been shown to stimulate DCs to produce IL-12 and IFNα/β, which subsequently induce Th1 responses (57), and IL-6, that in the presence of TGF-β, could induce a Th-17 response (58).

It is well known that, in order to evade the anti-tumoral immune response, tumor cells generate a regulatory tumor microenvironment. In this microenvironment, the T-cells express inhibitory molecules, such as PD-1, while the APC and the tumor cells express PD-L1 (24). Furthermore, the Treg population is increased in the tumor (59), and the antigen presentation to T cells is down-regulated (60). Accordingly, the microenvironment generated by the tumor model used in our study induced the expression of PD-1 in T-cells and of PD-L1 in APC and tumor cells; it also increased the percentage of Treg cells. When mice were vaccinated with the anti-DEC-205:16E5 conjugate, 70% controlled the tumor growth through the action of the E5-specific memory T cells. However, in 30% of the mice, these cells only delayed the tumor growth and eventually, the regulatory tumor microenvironment overcame this response killing the mice by day 72. In these mice’s tumors, T cells expressed PD-1, whereas APC and tumor cells expressed PD-L1, which could explain their failure to control the growth of the tumor. Moreover, this could explain the recovery of the expression of the 16E5 messages in the tumor cells by day 72.

It is likely that in the protected mice immunized with the anti-DEC-205:16E5 conjugate, the CD8^+^ T cells were functioning efficiently as effector cells, due to the effective T cell help provided by the 16E5-specific CD4^+^ T cells, which allowed them to eliminate the tumor cells. Previous studies have shown that the activation of the DCs by CD4^+^ T cells induces a strong cytotoxic program in the CD8^+^ T cells, including the down-regulation of inhibitory molecules such as PD-1 (61, 62). This effect depends on the interaction between CD70 in the DCs and CD27 in the CD8^+^ T cells. Depletion of the CD4^+^ T cells previous to the immunization with the anti-DEC-205:16E5 conjugate could provide more information on this issue.

To achieve 100% protection in mice vaccinated with the anti-DEC-205:16E5 conjugate, it may be necessary to increase the dose and/or the number of inoculations in order to generate a higher level of memory T cells with full effector functions. Alternatively, vaccination could be combined with immunotherapy. The axis PD-1/PD-L1 has been targeted successfully in malignant tumors, such as melanoma and lung cancer, by using mAbs in the so-called immune checkpoint therapy (63, 64). The fact that TILs present in the tumors from mice immunized with the anti-DEC-205:16E5 conjugate expressed PD-1 suggests that an immune checkpoint therapy could improve the survival of such mice. As expected, 5 doses of an anti-PD-1 mAb delayed the mice’s death for more than 20 days. Therefore, it follows that checkpoint therapy combined with vaccination improves the mice’s survival. However, further studies are necessary to find the best balance between vaccination and immunotherapy to achieve full protection.

Finally, we found that targeting the HPV16 E5 protein to DCs, in a therapeutic protocol of vaccination, induces a strong protection against E5-expressing tumor cells. Moreover, it was demonstrated that such protection is T cell-dependent and that an anti-PD-1 checkpoint therapy improves it. Since 16E5 is predominantly expressed in the early stages of HPV-induced CC, targeting E5 to DCs in humans could be an alternative for future immunization protocols in order to prevent and even eliminate these HPV-associated tumors.

## Data Availability Statement

The raw data supporting the conclusions of this article will be made available by the authors, without undue reservation.

## Ethics Statement

The animal study was reviewed and approved by the Ethics Committee of the National Institute of Public Health.

## Author Contributions

OB-G, FE-G., and LG-X conceived the study. OB-G carried out the immunizations and participated in the rest of the experimental techniques employed and drafted the manuscript. AP-S participated in RNA purification from tumor cells. MM-G participated in the characterization of the mAb-Ag conjugates. VV-G performed the qRT-PCR assays. VB-M participated in the establishment of the tumor model. RL-L participated in obtaining tumor cells and in the flow cytometry analysis. B-LC designed and supervised the experiments of the immune response characterization and the treatment of mice with mAbs. FE-G and LG-X directed the project and supervised the manuscript preparation. All authors participated in data interpretation. All authors contributed to the article and approved the submitted version.

## Funding

This work was supported by grants from SEP-CONACYT (No. 167265) and SEP-PRODEP (Redes Tematicas de Colaboracion de CA, 2015). OB-G was a Postdoctoral fellow of the Graduate Science Program, INSP, and was supported by CONACyT.

## Conflict of Interest

The authors declare that the research was conducted in the absence of any commercial or financial relationships that could be construed as a potential conflict of interest.
