# Supplementary material for: Induction of Therapeutic Protection in an HPV16-Associated Mouse Tumor Model Through Targeting the Human Papillomavirus-16 E5 Protein to Dendritic Cells
Source: Front Immunol. 2021 Feb 25;12:593161. doi: 10.3389/fimmu.2021.593161 (PMC7947241; doi:10.3389/fimmu.2021.593161)
Supplement: Supplementary file 1 [file DataSheet_1.pdf]

Supplementary Figure 1

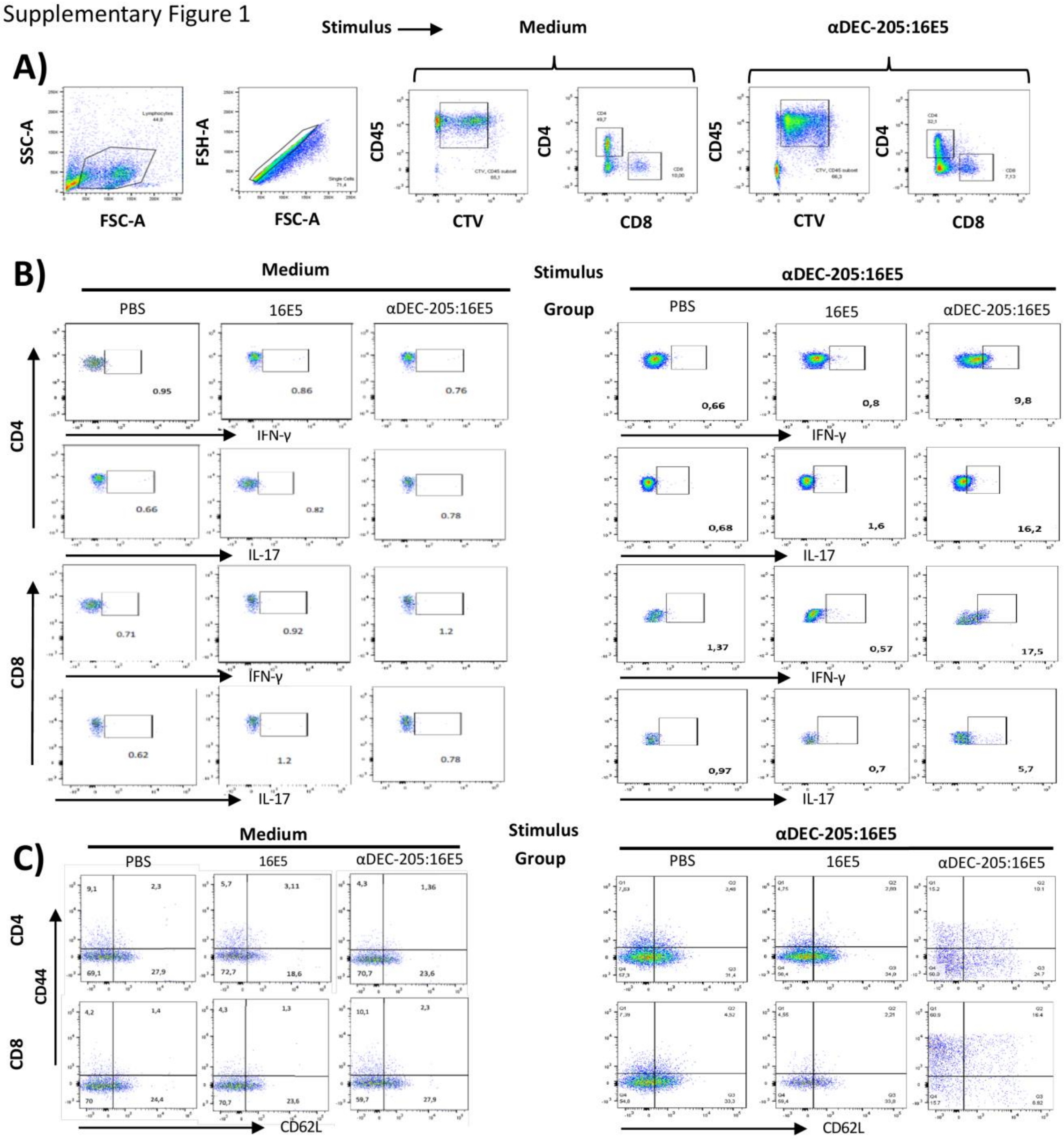

Supplementary Figure 1.- Dot plots of the flow cytometry analysis shown in Figures 3A and B and Figure 4A. Mice were inoculated with $5x10^5$ BMK-16/myc tumor cells and immunized s.c. with anti-DEC-205:16E5, free 16E5 or PBS in the presence of Poly I:C, using the therapeutic model. After 30 days of tumor cell inoculation, cells from the DLNs were labelled with celltrace violet (CTV) and stimulated *in vitro* with anti-DEC-205:16E5, anti-DEC-205:VP6 (as irrelevant antigen) or medium alone. After 7 days of stimulation, cells were collected, stained with a battery of mAbs specific for different T cell markers and cytokines coupled with fluorochromes, and analyzed by flow cytometry. Only the dot plots of cells stimulated with medium and anti-DEC-205:16E5 are shown since the stimulation with anti-DEC-205:VP6 gave similar results as medium alone. **(A)** Gattings used. **(B)** Expression of IFN-γ and IL-17 in $CD4^+$ and $CD8^+$ T cells. **(C)** Memory $CD4^+$ and $CD8^+$ T cells.

Supplementary Figure 2

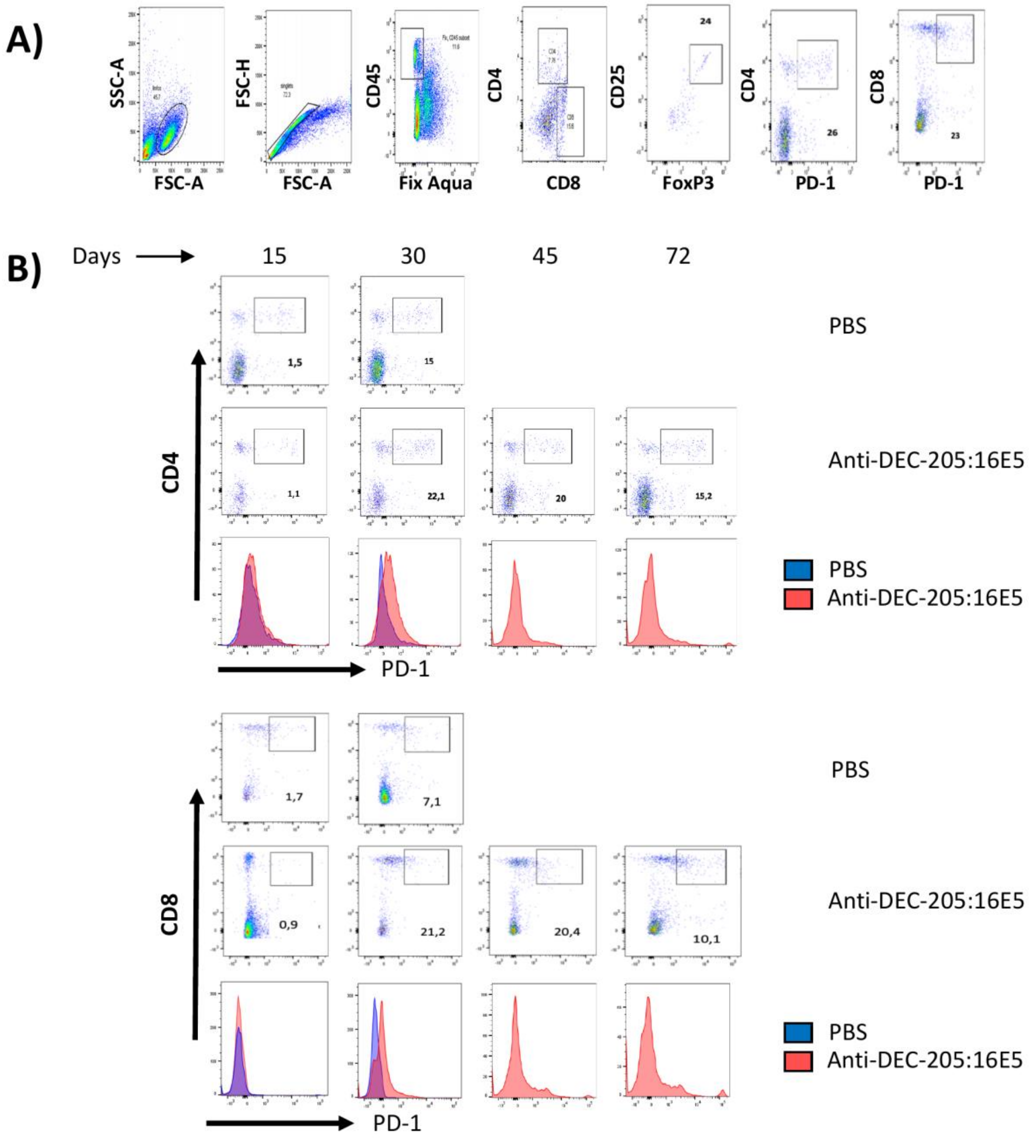

Supplementary Figure 2.- Dot plots of the flow cytometry analysis shown in Figures 5B and C. Mice were inoculated with $5x10^5$ BMK-16/myc tumor cells and immunized s.c. with PBS or anti-DEC-205:16E5 in the presence of Poly I:C, following the therapeutic model. Fifteen, 30, 45 and 72 days after tumor cell inoculation, TILs were obtained and stained with different mAbs coupled to fluorochromes and analyzed by flow cytometry. **(A)** Gattings used. **(B)** Expression of PD-1 in $CD4^+$ and $CD8^+$ T cells.

Supplementary Figure 3

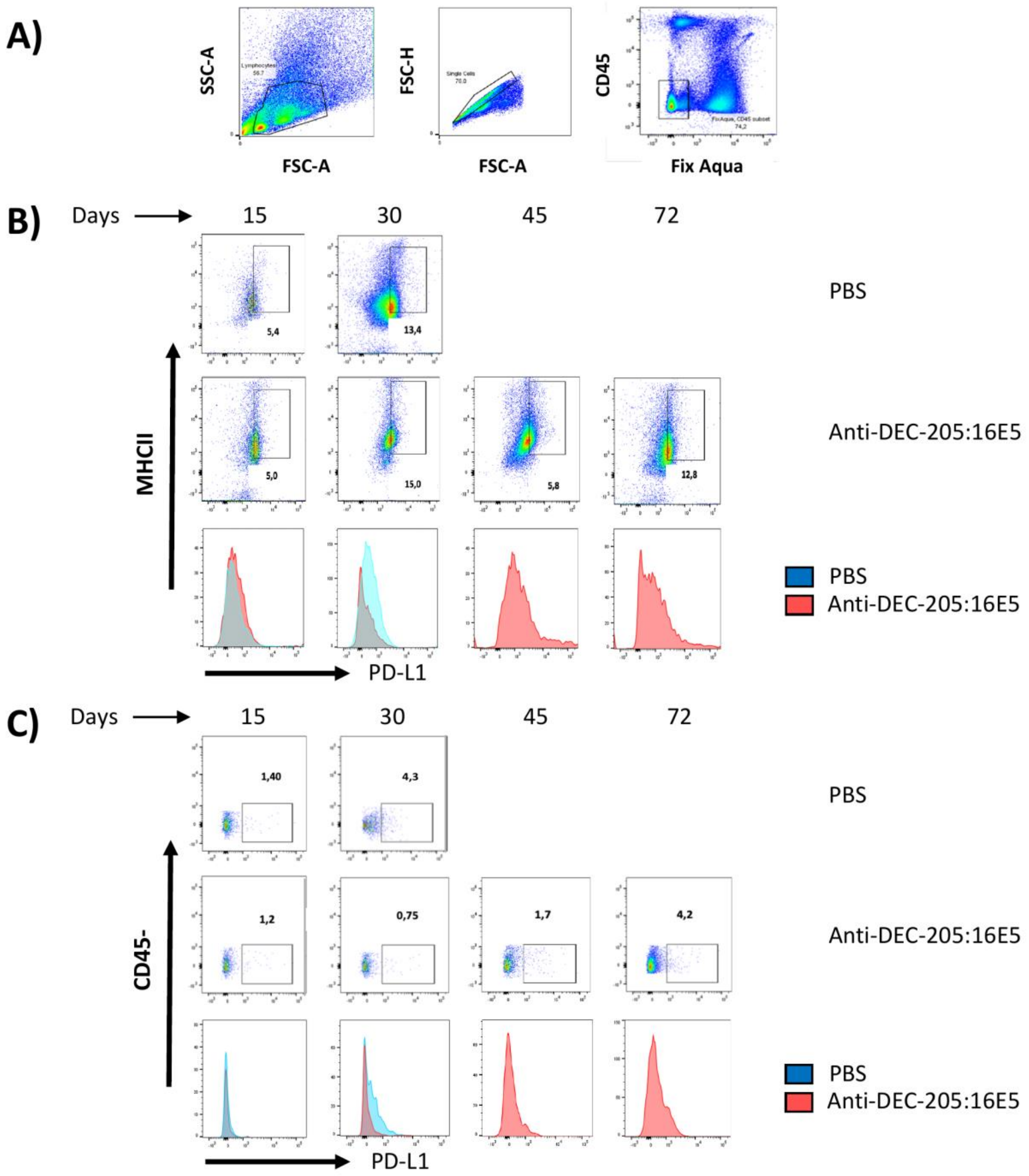

Supplementary Figure 3.- Dot Plots of the flow cytometry analysis shown in Figure 5D. Mice were inoculated with $5x10^5$ BMK-16/myc tumor cells and immunized s.c. with PBS or anti-DEC-205:16E5 in the presence of Poly I:C, following the therapeutic model. Fifteen, 30, 45 and 72 days after tumor cell inoculation, the cells from the disaggregated tumors were stained with different mAbs coupled to fluorochromes and analyzed by flow cytometry. **(A)** Gattings used. **(B)** Expression of PD-L1 in MHC II^+ cells. **(C)** Expression of PD-L1 in $CD45^-$ cells.

.
